# The Importance of Breast-Feeding

**Published:** 1920-11-06

**Authors:** 


### THE PUBLIC WELL-BEING.

### The Importance of Breast-Feeding.

One of the most interesting chapters in the maternity and child welfare section of the chief medical officer's report, some extracts from which we were able to publish last week, is that which deals with breast-feeding. It is a truism to say that breast-feeding is the safest, cheapest, and most satisfactory way of feeding the infant, at least up to nine months of age. This is generally admitted and believed, says Dr. Janet Campbell, but even yet for various reasons is not sufficiently practised. The mother's own milk is the only wholly satisfactory food for the infant, and there is no perfect substitute for it. It may be possible to prepare an artificial food chemically equivalent to breast milk, but such a food will still lack the proper supply of necessary food factors essential for healthy growth.

Breast-feeding is by no means always a simple and easy matter. Initial difficulties often arise, Dr. Campbell admits, which discourage the mother and in the absence of sensible practical advice may lead her to assume that she is physically unable to nurse her baby. "Unfortunately, the medical practitioner has relatively seldom made a special study of breast-feeding, and it is too often left to the monthly nurse or midwife to give advice in this matter. Unless the nurse has been well trained in the hygiene and management of breast-feeding she may be ready to despair of success before an adequate trial has been given. She may be ignorant of weaning difficulties, and may even prefer to suggest at the outset the adoption of artificial- instead of breast-feeding." Once the child is weaned the harm is done and cannot usually be remedied as far as that particular baby is concerned.

The old-fashioned custom of two-hourly feeding and night feeding placed a great strain, it is pointed out, upon the mother's health, strength, and patience. It occupied a very large portion of her time, and it was not surprising that she was not always able or willing to devote herself so completely to the service of the infant as the frequent feeding involved. Even if breast-feeding was established, there was a not unnatural tendency to prescribe occasional artificial feeds for the convenience and comfort of the mother. Once this habit was commenced it was easy gradually to increase the artificial- at the expense of the breast-feeding, especially when the infant discovered that the bottle was less trouble to take than the breast, and therefore showed a disposition to refuse the latter.

Modern teaching suggests that under the older method the infant was often over-fed, and its digestive system subjected to injurious and unnecessary strain. It is now generally accepted that feeding should be at intervals of not less than three hours from the time of birth onwards, and that the interval between the meals should be increased to four hours as soon as possible, no food being given at night between the hours of 10 P.M. and 6 A.M. Thus both mother and child have reasonable rest, and, from the mother's point of view, breast-feeding loses much of its inconvenience and fatigue.

Dr. Campbell urges the supreme importance of bringing to the notice of mothers and the community in general the full advantage of breast-feeding. "Doctors, midwives, nurses, and health visitors," she says, "should be thoroughly conversant with the practical aspect of the subject, and so able to give useful advice which will carry weight with the mother and convince her that it is not only right and proper to nurse her own baby, unless there are valid reasons to the contrary, but that the duty will not impose upon her an unduly heavy burden. It should be regarded as a matter for pride and congratulation when a mother, *whatever her position in life*, is able to nurse her baby herself, while the mother who has to resort to artificial methods of feeding should be looked upon as deserving of commiseration."

This point of view is in line with the administrative details given in the first report of the Ministry of Health on the work done in connection with maternity and child welfare. The county and borough councils are urged to appoint assistant medical officers, if possible women, whose special duty will be to supervise this work. Already a hundred such officers have been appointed, and there are over 3,000 health visitors wholly or partly engaged in the work. The policy, however, is that alternately the visiting of infants and expectant mothers should be assigned to trained midwives. The number of municipal welfare centres has been increased from 396 in 1916 to 1,061 in 1920; the number of voluntary centres has advanced from 446 to 693, and it is satisfactory to know that the voluntary centres are cordially co-operating with the local authorities.

